# DNA Methylation Associates With Clinical Courses of Atypical Meningiomas: A Matched Case–Control Study

**DOI:** 10.3389/fonc.2022.811729

**Published:** 2022-03-09

**Authors:** Matthias Millesi, Alice Senta Ryba, Johannes A. Hainfellner, Thomas Roetzer, Anna Sophie Berghoff, Matthias Preusser, Gerwin Heller, Erwin Tomasich, Felix Sahm, Karl Roessler, Stefan Wolfsberger

**Affiliations:** ^1^ Department of Neurosurgery, Medical University of Vienna, Vienna, Austria; ^2^ Comprehensive Cancer Center, Central Nervous System Unit, Medical University of Vienna, Vienna, Austria; ^3^ Division of Neuropathology and Neurochemistry, Department of Neurology, Medical University of Vienna, Vienna, Austria; ^4^ Department of Internal Medicine I/Oncology, Medical University of Vienna, Vienna, Austria; ^5^ Department of Neuropathology, Institute of Pathology, Ruprecht-Karls-University Heidelberg, Heidelberg, Germany; ^6^ Clinical Cooperation Unit (CCU), German Cancer Consortium (DKTK), German Cancer Research Center (DKFZ) Heidelberg, Heidelberg, Germany

**Keywords:** meningioma, atypical, DNA methylation, Wnt pathway, prognosis

## Abstract

**Background:**

Accounting for 15–20% of all meningiomas, WHO grade II meningiomas represent an intermediate group regarding risk of tumor recurrence. However, even within this subgroup varying clinical courses are observed with potential occurrence of multiple recurrences. Recently, DNA methylation profiles showed their value for distinguishing biological behaviors in meningiomas. Therefore, aim of this study was to investigate DNA methylation profiles in WHO grade II meningiomas.

**Methods:**

All patients that underwent resection of WHO grade II meningiomas between 1993 and 2015 were screened for a dismal course clinical course with ≥2 recurrences. These were matched to control cases with benign clinical courses without tumor recurrence. DNA methylation was assessed using the Infinium Methylation EPIC BeadChip microarray. Unsupervised hierarchical clustering was performed for identification of DNA methylation profiles associated with such a dismal clinical course.

**Results:**

Overall, 11 patients with WHO grade II meningiomas with ≥2 recurrences (Group dismal) and matched 11 patients without tumor recurrence (Group benign) were identified. DNA methylation profiles revealed 3 clusters—one comprising only patients of group dismal, a second cluster comprising mainly patients from group benign and a third cluster comprising one group dismal and one group benign patient. Based on differential methylation pattern associations with the Wnt and the related cadherin signaling pathway was observed.

**Conclusion:**

DNA methylation clustering showed remarkable differences between two matched subgroups of WHO grade II meningiomas. Thus, DNA methylation profiles may have the potential to support prognostic considerations regarding meningioma recurrence and radiotherapeutic treatment allocation after surgical resection.

## Introduction

More than 30% of intracranial CNS tumors represent meningiomas which are considered slowly growing tumors in the majority of cases (1, 2). Apart from small or surgically not amenable meningiomas that are commonly treated with radiosurgery, the aim of modern meningioma treatment is complete surgical resection, including the underlying dura and infiltrated bone (3, 4). The extent of resection (EOR) as classified by the Simpson grading scale has been found to correlate with recurrence (3, 5).

To describe the biological tumor behavior, the World Health Organization (WHO) has tried to classify meningiomas by a three-tiered system based on histopathological criteria (6, 7). In this sense, WHO grade I meningiomas are considered benign and usually do not require further treatment after surgical resection (4, 7). On the other end, WHO grade III (anaplastic or malignant) meningiomas are characterized by a very aggressive clinical behavior with a high recurrence rate and therefore adjuvant radiation therapy is mandatory (4, 7).

Further, there is an intermediate class of meningiomas with a biological behavior between benign and anaplastic. As early as 1939, Cushing and Eisenhardt described these as a “low-malignancy subgroup” of malignant meningiomas and it was not before 1993 that the WHO coined the term “atypical” meningioma grade II (6, 8). Initially, the criteria were not very well defined, however, in a continuous search to better define grade II meningiomas with aggressive biological behavior, the histopathological criteria have been refined over the revisions of the WHO classification. In this sense, they were defined as between 4 and 20 mitoses per 10 high-power fields and histopathologic characteristics such as increased cellularity, high nucleus/cytoplasm ratio, patternless or sheet-like growth and spontaneous tumor necrosis in 2000 and evidence of brain invasion was added in 2016 (6–9). Despite these refinements, the biological behavior of WHO grade II atypical meningiomas remains unpredictable; even after gross-total resection the clinical course can vary between benign (long-term survival after a single surgical resection) and dismal (repeated recurrences despite multiple treatments and the patient eventually succumbing to the disease).

The current treatment recommendation of adjuvant radiotherapy for *incompletely* resected grade II meningiomas is generally accepted (4). However, there is an ongoing debate if patients should also receive radiotherapy after *complete* surgical resection of a grade II meningioma to prevent a possible dismal course of the disease, because of the potential side-effects of unnecessary irradiation (4, 10). Therefore, there is a yet unmet need for parameters that predict the biological behavior of a given WHO II atypical meningioma.

With the advent of molecular diagnostics, attempts have been made to better classify the meningiomas in this respect. Recently, the methylation status of the DNA also got into the focus of interest for potentially indicating such a different biological behavior (11–13). DNA methylation that mainly occurs at CpG dinucleotides leads to either loss of gene expression (hypermethylation) or gene activation (hypomethylation).

The aim of this study was to investigate DNA methylation of WHO grade II meningiomas with dismal course and compare to a matched control group with benign biological behavior whose clinical, radiological and histopathological characteristics are indistinguishable. The hypothesis is that the differential methylation pattern signature significantly differs between the two groups and may potentially be of prognostic value.

## Material and Methods

All adult patients who underwent resection of a newly diagnosed meningioma at our institution between 1993 and 2015 were screened for grade II classified by WHO criteria of the time of surgery. This starting point was chosen because of the inclusion of grade II in the WHO classification from 1993 onwards. The end point 2015 was chosen to allow a possible minimum follow-up time of 5 years. Ethics approval was waived due to the retrospective nature of this study and the sole evaluation of existing pathologic specimens only.

Of all selected patients with WHO grade II meningioma, a retrospective chart and preoperative imaging review was performed, if available. All treatments, surgical and adjuvant, were recorded.

The exact location of each tumor, its size and volume, shape, surface irregularity, edema and EOR were determined.

Location was stratified as convexity (frontal, central, parietal, occipital, temporal), parasagittal (anterior, middle, posterior third), falcine (anterior, middle, posterior third), skull base location, tentorial, and ventricular.

Size and volume were determined as maximum meningioma diameter and volumetric assessment on preoperative imaging, respectively.

The EOR was classified according to the Simpson classification as documented in the surgical reports and confirmed by postoperative imaging. Then, the EOR was stratified as “gross-total” (Simpson 1–3) versus “partial” (Simpson 4–5) as previously described (4).

Exclusion criteria for this study represented treatment for an already recurring WHO grade II meningioma or any prior treatment for an intracranial meningioma and surgical resection of a meningioma other than WHO grade II.

### Case–Control Selection

Of all eligible patients, a matched case–control series based on the clinical course was then compiled. The case cohort consisted of patients that showed a prominent dismal clinical course (Group dismal) with multiple tumor recurrences despite all available treatments (defined as ≥2 recurrences in the observation period). The time to recurrence in each patient was noted and defined as the date of the next treatment. Additionally, the status at the end of the observation period, alive or dead, and the cause of death were noted.

The control group consisted of patients with a benign course (Group benign) of their WHO grade II meningioma with one surgical treatment only, without adjuvant radiation therapy and without recurrence in the observation period of at least 5 years. Case–control matching was performed with age at presentation, tumor location, tumor size, EOR and Ki-67 proliferation index.

For analyzing the Ki-67 proliferation index, this was stained using the MIB-1 antibody (1:200, Dako Cytomation, M7240) using a Dako autostainer system. In a next step, these were then digitized with a Hamamatsu NanoZoomer 2.0 HT slide scanner and automated MIB-counting was performed using a similar image analysis algorithm pipeline as previously published (14). Therefore, a custom MATLAB-script (R2017b, MathWorks) with Phansalkar thresholding was used for identification of DAB-stained and hematoxylin-stained cells (15). The quotient of DAB+-cells to hematoxylin+-cells was calculated to obtain the average Ki-67 cell proliferation index for the whole slide. The resulting Ki-67 proliferation index was then compared between the two groups. Furthermore, a heatmap for cell proliferation was calculated by the quotient of DAB+/hematoxylin+ cell-centroids for each pixel in the whole slide. Based on this heatmap, a hotspot was defined for analysis above the 99th percentile and the resulting Ki-67 proliferation index for the hotspot was compared separately between the two groups.

### DNA Extraction and Sodium Bisulfite Conversion

Genomic DNA (gDNA) was extracted from 3 to 5 × 10 µm slices of formalin-fixed, paraffin-embedded (FFPE) meningioma specimens using the Maxwell FFPE Plus Kit (Promega, Madison, Wisconsin, USA). A total of 500 ng of extracted gDNA was first sodium bisulfite converted using the EZ DNA Methylation™ Kit (Zymo Research, Irvine, CA, USA) and then repaired using the Infinium HD FFPE DNA Restore Kit (Illumina, San Diego, California, USA) according to the manufacturers’ instructions.

### DNA Methylation Profiling

DNA methylation of more than 850,000 CpG sites was assessed using Infinium Methylation EPIC BeadChip microarrays (Illumina, San Diego, California, USA). In brief, sodium bisulfite treated gDNA was amplified and enzymatically fragmented followed by microarray hybridization as recommended by the manufacturers. After washing and staining, microarrays were analyzed on an iScan device (Illumina, San Diego, California, USA).

Following this, meningiomas with similar DNA methylation profiles were grouped together by unsupervised hierarchical clustering. These clusters were then compared to the proposed Heidelberg classifier by Sahm et al. (ben-1, ben-2 ben-3, int-A, int-B and mal) and also the combined methylation classes (benign, intermediate and malignant, available from MolecularNeuroPathology.org) (12).

### Statistical Analysis

For statistical analyses, the matched cohorts of patients were compared using a chi-square test for categorical variables such as gender, tumor location and EOR. Continuous variables such as age, tumor size and the mitotic index were analyzed using a Mann–Whitney-U-test. A two-sided p-value <0.05 was considered statistically significant.

For analyses of the DNA methylation profiles, raw microarray data (.idat files) were imported into R (version R 3.6.1, R Foundation for Statistical Computing, Vienna, Austria) using the ChAMP package for initial quality control and calculation of differential DNA methylation (16). Probes with a detection p-value >0.01 in one or more samples, with a bead count <3 in at least 5% of all samples, non-CpG probes, probes with SNPs and, probes which align to multiple locations and sex chromosome-specific probes were excluded from subsequent analyses. Data normalization was performed using the SWAN algorithm followed by COMBAT batch correction (17). Differential methylation between groups was defined as |beta value difference| >0.1 and adjusted *P*-value (Benjamini–Hochberg method, FDR) <0.01. To validate differential methylation results, data analyses were repeated using the R package RnBeads and only CpG sites found to be differentially methylated in both settings were used for further evaluation (18). KEGG pathway and gene ontology (GO) enrichment analyses were done using the WebGestalt tool (19).

## Results

Of all 1,675 patients who underwent surgical resection of a newly diagnosed meningioma, an atypical WHO grade II meningioma was diagnosed in 179 patients (10.7%). Of these, 11 patients were allocated to group dismal as they showed such a salient dismal clinical course with ≥2 recurrences. At the end of the observation period (median follow-up of 98 months, range 50–150 months), 9 of these patients had died of their meningioma and one patient still suffers from active disease.

**Figure 1:**
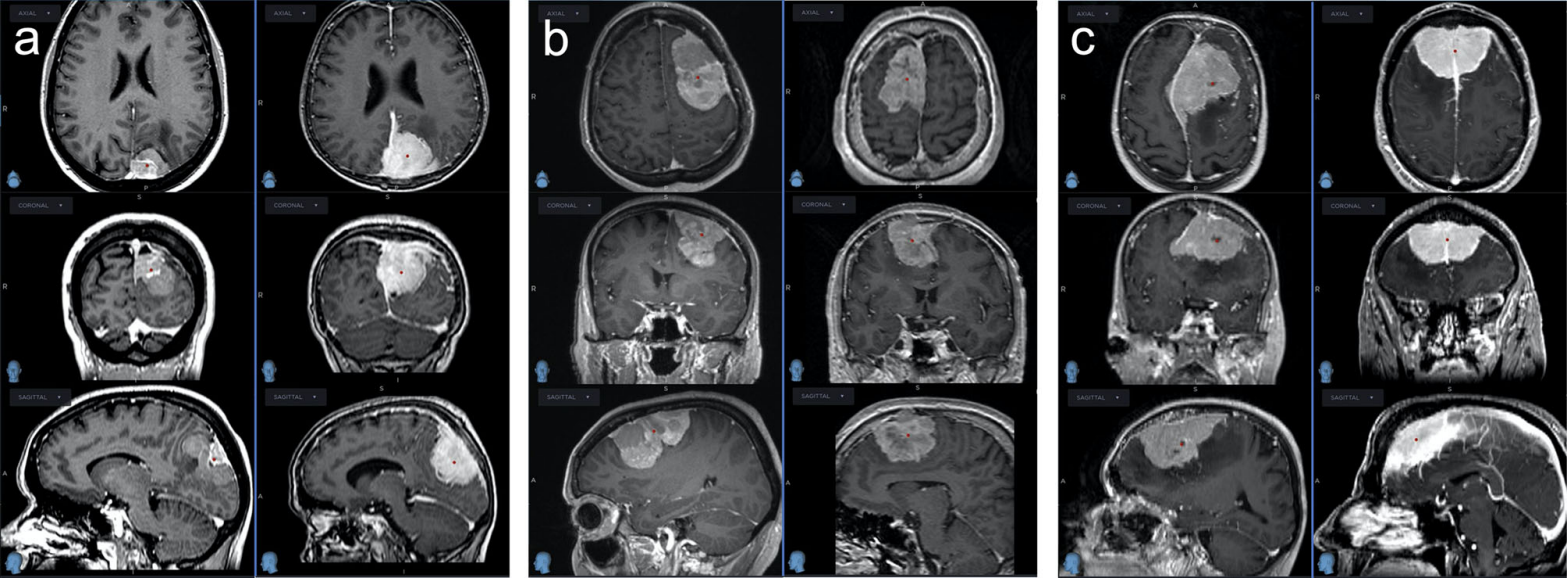
Three pairs of WHO grade II meningiomas matched for tumor size, location and mitotic index; the left rows with dismal clinical course, the right rows with benign clinical course, none had radiotherapy after GTR. **(A)** Left column: This meningioma recurred 1.2 years after GTR at multiple sites and was treated with 2 re-operations and 2 radiosurgeries. Right column: Following GTR, this meningioma never recurred after a follow-up of 17 years. **(B)** Left column: After initial GTR, this meningioma recurred locally after 2 years at multiple sites. It was treated with 6 operations and multiple radiotherapeutic options before the patient finally succumbed to the disease 8 years after diagnosis. Right column: This meningioma never recurred after GTR within the follow-up of 6.5 years. **(C)** Left column: This meningioma first recurred 2.5 years after GTR and was reoperated multiple times before the patient finally died from the disease after 9 years. Right column: This meningioma never recurred within a follow-up duration of 9 years.

On the opposite, the matched control cohort (group benign) comprised 11 patients of the same tumor location, size, EOR and mitotic index. These patients did not show any recurrences of their first identified tumor in a median follow-up period of 114 months (78–246 months). Comparing all characteristics for comparison, no variable but age showed a statistically significant difference between the groups. For an overview and comparison of patient characteristics see **Tables 1**, **2**. A number of illustrative cases are given in **Figure 1**.

**Table 1:** Patient characteristics.

	N	%
number of included patients	22	100
median age	64 years (19–73 years)	
female: male ratio	1: 1	
tumor location		
parasagittal/parafalcine	18	82
sphenoid wing	2	9
middle fossa floor	2	9
EOR		
Simpson I	11	50
Simpson II	9	40
Simpson III	1	5
Simpson IV	1	5

EOR, extent of resection.

**Table 2:** Comparison of patient characteristics.

	Group D n = 11	Group B n = 11	p-value
Age* (years)	61 (45–74)	50 (19–67)	**0.023**
location (non-skull base, %)	81	81	0.716
tumor size* (cm)	5.0 (3.0–7.0)	5.0 (2.5–8.0)	0.742
EOR (GTR, %)	91	91	1.000
MIB overall* (%)	2.8 (0.3–26.5)	1.8 (0.4–9.2)	0.577
MIB hotspot* (%)	8.5 (2.2–36.0)	6.5 (2.7–18.0)	0.577

EOR, extent of resection; GTR, gross total resection.

^*^median (range).

The bold value signify the statistical significant difference in the characteristic age.

### DNA Methylation Profiling of Meningioma Patients

**Figure 2:**
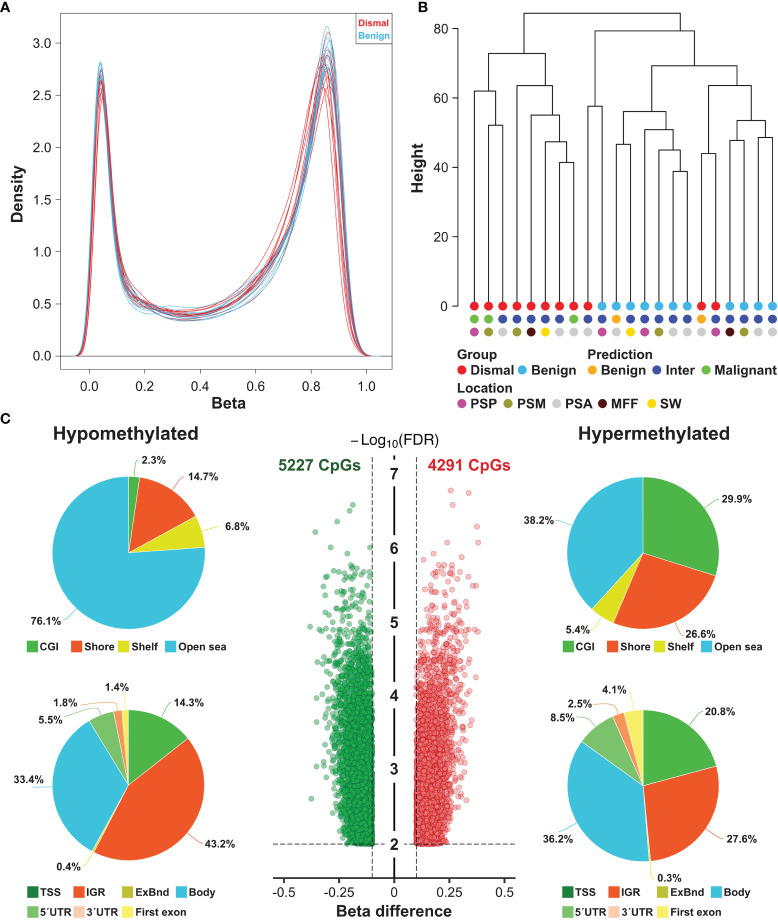
Large scale DNA methylation analyses of tumor specimens from meningioma patients. **(A)** Density plot showing the bimodal ß-value distribution of 688,310 probes after normalization and background correction. **(B)** Unsupervised hierarchical clustering of meningioma samples based on methylation of 688,310 probes revealed 3 distinct clusters (one consisting only of dismal cases, a second one comprising mostly benign cases and a small third cluster with one benign and one dismal case. These were also compared to the previously proposed methylation classes (benign, intermediate and malignant). Inter, intermediate, PSP, parasagittal posterior third; PSM, parasagittal middle third; PSA, parasagittal anterior third; MFF, middle fossa floor; SW, sphenoid wing. **(C)** Number and location of differentially methylated CpG sites (DMP) between meningioma patients showing a salient dismal clinical course (*N* = 11) and matched control individuals (*N* = 11). Each dot represents a unique DMP (green hypomethylated; red, hypermethylated). The location of these DMPs relative to CpG islands (CGI) and genomic features are shown in the upper and lower pie charts, respectively. TSS, transcription start site; IGR, intergenic regions; UTR, untranslated region.

To determine if the methylome of group dismal differs from the methylome of the matched control cohort (group benign), we performed Illumina methylation EPIC microarray analyses. Filtering of low quality and sex chromosome specific probes yielded a total of 688,310 remaining probes for further statistical evaluation. Singular Value Decomposition (SVD) analysis indicated that a significant variation of DNA methylation within the whole study cohort was associated with array batches and patients’ age (**Supplementary Figure S1A**). In addition, although data were normalized, a strong variation within our dataset was seen in beta value density (**Supplementary Figure S1B**). Thus, methylation data were batch corrected resulting in a highly comparable bimodal distribution of beta value density across all 22 samples analyzed (**Figure 2A**). Unsupervised hierarchical clustering of the whole study population based on 688,310 adjusted beta values was performed to test for large scale DNA methylation heterogeneity within the cohort.

According to the methylation signature, three separate clusters were identified (**Figure 2B**): One cluster contained only patients of group dismal (8 of 11 patients (73%)), one small cluster with 1 patient of group dismal and 1 patient of group benign, and a third cluster containing 10 cases (91%) of group benign and only 2 cases of group dismal.

These methylation profiles were then compared to the proposed combined methylation classes by the Heidelberg classifier by Sahm et al. (available from MolecularNeuroPathology.org) (12). Based on the predicted combined meningioma methylation classes, all their malignant cases were in our group dismal. Interestingly, two cases would be classified as benign (one case ben-1, one case ben-2). However, one of these cases was in our group dismal and one case in our group benign. Overall, the majority of all cases (17 patients, 77%) was classified as intermediate (int-A n = 16, int-B n = 1), so these methylation classes did not clearly affect sample clustering. Similarly, location of the meningioma also did not affect sample clustering (**Figure 2B**).

These data demonstrate that the methylome of meningioma patients with dismal clinical course substantially differs from the methylome of control subjects.

### Differential Methylation Analysis

**Figure 3:**
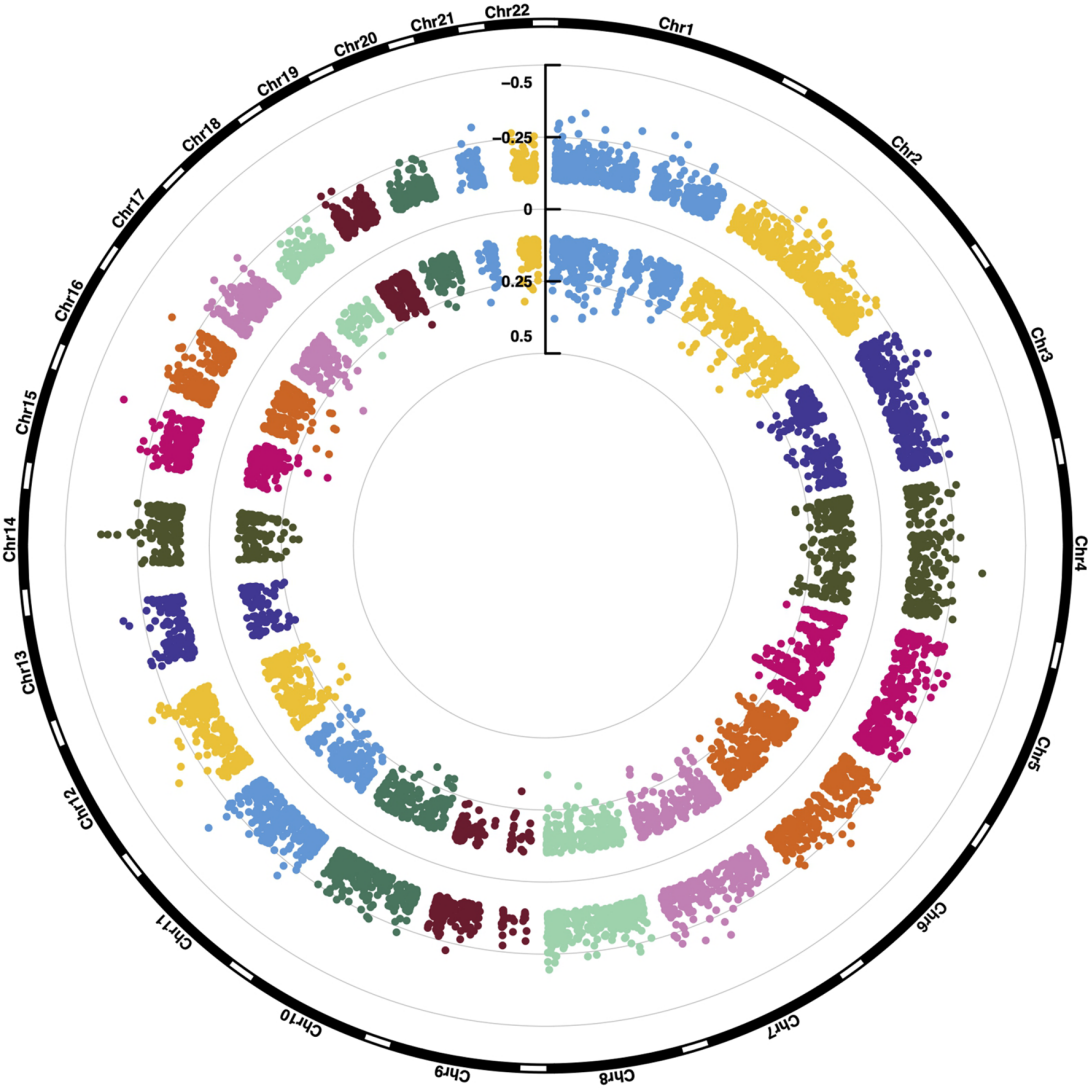
Chromosomal location of 9,518 differentially methylated CpG sites. ß-value differences are shown.

In a next step, we tested for significant differences in methylation of the 688,310 CpG sites between group dismal and group benign. In total, the differential methylation pattern (DMP) comprised 9,518 CpG sites (1.4% of the CpG sites analyzed) between the two patient groups (FDR <0.01, |ß-difference| >0.1). While methylation of 5,227 DMPs was decreased, methylation of 4,291 DMPs was increased (**Figure 2C**). By mapping the DMPs to their genomic location within (island), around (shore and shelf) or between (open sea) CpG islands, we found an over-representation of hypomethylated non-CpG island DMPs (76.1%). The frequency of hypermethylated non-CpG island DMPs was lower (38.2%) with more than 50% of CGI/shore/shelf located DMPs (**Figure 2C**). The frequencies of hypomethylated and hypermethylated CpG island associated DMPs were 2.3 and 29.9% for CGIs, 14.7 and 26.6% for shore regions (2-kb regions flanking CpG islands), and 6.8 and 5.4% for shelve regions (2-kb regions of flanking shore regions), respectively. The locations of hypomethylated DMPs in the context of gene-associated regions were 33.4% in gene bodies, 43.2% in intergenic regions (IGR), 14.3% in transcriptional start sites, 5.5% in 5´ UTRs, 1.8% in 3´ UTRs, 1.4% in first exons and 0.4% in exon boundaries. These values are very similar for hypermethylated DMPs with the exception of a lower IGR frequency and a higher gene body/TSS frequency. The distribution of hypo- and hypermethylated DMPs over chromosomes is relatively even as shown in **Figure 3**.

### Functional Implication of Differentially Methylated Gene

**Figure 4:**
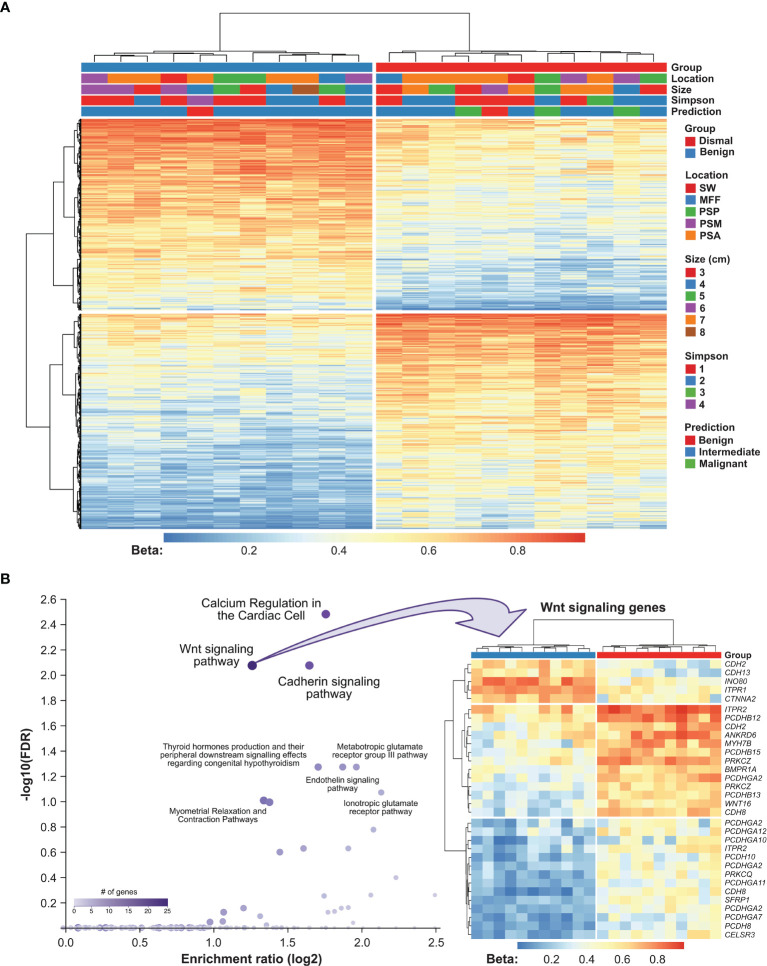
Functional implication of DMPs between meningioma patients showing a salient dismal clinical course and controls. **(A)** Heatmap showing methylation of 1,348 CpG sites in 22 meningioma samples. Patients’ characteristics, namely, tumor recurrence, meningioma location, size, EOR (according to Simpson grade) and methylation class prediction are displayed according to the color scheme shown at the right-hand side of the figure. Each column represents a unique patient sample and each row represents a unique CpG site. Heatmap colors reflect beta values representing the degree of methylation from low (blue) to high (red). No centering/scaling of beta values was performed. Simpson, Simpson grade; PSP, parasagittal posterior third; PSM, parasagittal middle third; PSA, parasagittal anterior third; MFF, middle fossa floor; SW, sphenoid wing. **(B)** Results from pathway enrichment analysis using WebGestalt software (left panel). Each dot represents a unique pathway. Heatmap summarizing methylation of Wnt signaling genes in 22 meningioma samples. Genes which appear repeatedly in the heatmap were affected by multiple DMPs. Heatmap colors reflect beta values representing the degree of methylation from low (blue) to high (red). No centering/scaling of beta values was performed.

To investigate the potential biological relevance of differential methylation between meningioma patients showing a salient dismal clinical course and controls, we further filtered our data for the strongest methylation differences defined by an adjusted *P*-value <0.01 and a |beta difference| of at least 0.2. 1,348 DMPs met these stringent selection criteria (635 hypo- and 713 hypermethylated; **Figure 4A** and **Supplementary Table S1**). Methylation of them was only associated with a dismal clinical course and no association with meningioma location, meningioma size, Simpson grade or predicted meningioma methylation class (MolecularNeuroPathology.org) was seen. The 1,348 DMPs were associated with 683 genes, which were used for further pathway enrichment analyses. We compared these 683 genes with deregulated methylation with a background list of the human genome and their relation to signaling pathways.

Among annotated pathways, the top hits were Wnt signaling pathway (FDR = 0.008) and the related cadherin signaling pathway (FDR = 0.008). The differential methylation pattern of Wnt signaling pathway members in our meningioma patient cohort is shown in **Figure 3B**. Furthermore, calcium regulation in the cardiac cell (FDR = 0.003), endoderm differentiation (FDR = 0.054) and G protein signaling pathways (FDR = 0.054; **Figure 4B**, **Supplementary Table S2**) were represented. Gene ontology enrichment analysis showed a significant over-representation of genes mainly involved in developmental processes, cell adhesion and cell differentiation (**Supplementary Table S3**).

## Discussion

Despite assumed complete tumor removal and similar radiologic and histopathologic appearance, the clinical courses of WHO grade II meningiomas can present in clinical practice in a very heterogeneous pattern: while some of these patients never develop tumor recurrence during their follow-up, others experience a very dismal clinical course with early and multiple tumor recurrences potentially leading to the death of patients. Additionally, there is an ongoing debate in the scientific community if patients that underwent complete resection of a WHO grade II meningioma should undergo watchful waiting or adjuvant radiotherapy (4, 20–23).

Several different prognosticators, namely, clinical–radiological, surgical or pathological characteristics have been investigated; however, no reliable consistent factors could be identified (24–31). Additionally, genetic analyses have revealed some data on mutations associated with the WHO grade in recent years but similarly to the aforementioned aspects, no clearly reliable indicators were detected (12, 32–35). In this sense, epigenetics and especially DNA methylation got in the focus of interest and based on the DNA methylation profile, classes or nomograms have been proposed that differ from the traditional WHO grading for a better prognostic estimation (11, 12, 35, 36). In this sense, 6 distinct groups of methylation classes (ben-1, ben-2, ben-3, int-A, int-B and mal) of meningiomas were identified in a study by Sahm et al. based on the methylation signature (12). These classes differed in their biological behavior and the frequency and time to tumor recurrence from the traditional WHO grading based on morphological characteristics. In contrast, a good overlap in progression-free survival within the group of benign (ben-1 through ben-3) compared to the intermediate (int-A and int-B) or the malignant (mal) could be observed with an even greater distinction from the progression-free survival of the traditional WHO groups (12). However, these analyses have been performed on a mixed cohort of meningiomas of all WHO grades and did not address the specific subgroup of WHO grade II meningiomas.

Therefore, the aim of this study was to investigate the value of DNA methylation analyses in more detail in this specific subgroup of WHO grade II meningiomas. Hence, a group of 11 patients was identified that showed a salient dismal course and underwent multiple surgeries for recurrent tumors and some even died of this disease from a large patient cohort of 179 WHO grade II meningiomas (group dismal). A control group of 11 cases matched for age, tumor location, tumor size, EOR and the mitotic index were noted that had a very benign clinical course without tumor recurrence after their initial resection was chosen (group benign).

### Potential Prognostic Value of the DNA Methylation Signature

In this matched case–control series of 22 patients unsupervised hierarchical clustering revealed 2 distinct clusters of our series after analysis of DNA methylation: One cluster containing only patients with a dismal clinical course and one cluster with mostly patients with a benign clinical course. A third small cluster contained 1 patient of group dismal and group benign, respectively. A comparison of these results to the open-source data for the proposed methylation classes by Sahm et al. (MolecularNeuropathology.org) revealed that the majority of our patients overall were found in the combined intermediate class. This finding is in line with the results of the previous study by Sahm et al. (12). However, as 3 meningiomas in the dismal cluster of our series were classified as malignant methylation class and two cases as benign instead of intermediate. This might be a result of the very selected patient cohort in this study based on the defined case–control design. On the other hand, although certainly associated with progression-free survival, the aspect that the proposed methylation classes might not give a perfect prognostic discrimination in the particular case of WHO grade II meningiomas could represent another potential explanation.

### Implication of Differential Methylation Patterns for Molecular Signaling Pathways

Besides the observed methylation clustering, pathway enrichment analyses revealed an overrepresentation of deregulated genes mainly for the Wnt signaling pathway (FDR <0.0001) and the related cadherin signaling pathway (FDR <0.0001, see **Figure 3B**). The Wnt signaling pathway and especially its deregulation has been shown to be involved in a variety of different tumors playing an important role in development and progression of differing cancers (37–39). Recently, this has also become of interest in the tumorigenesis of meningiomas (35, 38). Similarly, frequent loss of the related cadherin signaling pathway mediated by the canonical beta-catenin was also found in anaplastic meningiomas and reported in the recent literature (35).

### Future Outlook and Relevance of the Study

Despite the small sample size of this cohort, the molecular pathologic results of this study reveal remarkable differences between the two subgroups of distinct clinical behaviors of WHO II meningiomas.

However, future efforts should be directed towards repeating this analysis in a larger cohort of patients with WHO grade II meningiomas taking these diverse clinical courses into special account. As a consequence, if future larger studies confirm our findings, these methylation clusters may become useful for an estimation of patient prognosis, risk of meningioma recurrence and therefore potential treatment allocation after the initial surgical resection. Also, an investigation for potential associations of these DNA methylation profiles and genetic or chromosomal aberrations should be performed in future studies.

In addition, the observed association of the strongest methylation differences with the Wnt and related cadherin signaling pathway in those patients with a salient dismal clinical course might become of interest for novel chemotherapeutic agents.

### Limitations

The small sample size of 11 patients in the study cohort with such a dismal clinical course represents one important limitation of this study. Also, the investigated method has not been validated in an independent patient cohort. Hence, formulation of the clear prognostic value of these DNA methylation profiles needs confirmation in independent and larger study cohorts. Despite this aspect, the estimation of the EOR which is typically done by the treating surgeon in intracranial meningiomas according to the Simpson classification and therefore is subject to misinterpretation or missing out small tumor remnants within bone or surrounding tissue. To overcome this, a more standardized approach, namely, postoperative morphological imaging consisting of MRI or even metabolic imaging including positron emission tomography to detect small tumor residuals is warranted to better stratify the EOR.

### Conclusion

Methylation analysis was shown to correlate with clinical prognosis irrespective of the WHO grading system and is currently discussed as cooperation in the diagnostic approach. The clinical behavior of grade II atypical meningiomas may be unpredictable with tumors recurring repeatedly whereas others do not—despite being clinically indistinguishable.

The presented DNA methylation clustering shows remarkable differences between two matched subgroups of different clinical behavior of WHO II meningiomas. DNA methylation profiles may have the potential to support prognostic considerations regarding meningioma recurrence and radiotherapeutic treatment allocation after surgical resection and should be investigated in clinical, prospective studies.

## Data Availability Statement

The original contributions presented in the study are included in the article/**Supplementary Material**. Further inquiries can be directed to the corresponding author.

## Ethics Statement

Ethical review and approval was not required for the study on human participants in accordance with the local legislation and institutional requirements. Written informed consent for participation was not required for this study in accordance with the national legislation and the institutional requirements.

## Author Contributions

Conceptualization: MM, ASR, and SW Data acquisition: MM, ASR, and SW Data and statistical analyses: MM, ASR, TR, ASB, MP, GH, ET, and SW Manuscript preparation: MM, ASR, and SW. Writing—Review and editing MM, ASR, JAH, TR, ASB, MP, GH, ET, FS, KR, and SW. Study supervision: SW. All authors listed have made a substantial, direct, and intellectual contribution to the work and approved it for publication.

## Conflict of Interest

AB has received research support from Daiichi and Roche and honoraria for lectures, consultation or advisory board participation from Roche, Bristol-Myers Squibb, Merck, Daiichi Sankyo, AstraZeneca and also travel support from Roche, Amgen and AbbVie. MP has received honoraria for lectures, consultation or advisory board participation from the following for-profit companies: Bayer, Bristol-Myers Squibb, Novartis, Gerson Lehrman Group (GLG), CMC Contrast, GlaxoSmithKline, Mundipharma, Roche, BMJ Journals, MedMedia, Astra Zeneca, AbbVie, Lilly, Medahead, Daiichi Sankyo, Sanofi, Merck Sharp & Dome, Tocagen, Adastra, Gan & Lee Pharmaceuticals. GH has received research funding from Bristol-Myers Squibb and MSD. SW is an educational consultant and a technological advisory board member of Medtronic Navigation. FS has received honoraria from Illumina.

The remaining authors declare that the research was conducted in the absence of any commercial or financial relationships that could be construed as a potential conflict of interest.

## Publisher’s Note

All claims expressed in this article are solely those of the authors and do not necessarily represent those of their affiliated organizations, or those of the publisher, the editors and the reviewers. Any product that may be evaluated in this article, or claim that may be made by its manufacturer, is not guaranteed or endorsed by the publisher.
