# Supplementary material for: DNA Methylation Associates With Clinical Courses of Atypical Meningiomas: A Matched Case–Control Study
Source: Front Oncol. 2022 Mar 9;12:811729. doi: 10.3389/fonc.2022.811729 (PMC8959647; doi:10.3389/fonc.2022.811729)
Supplement: Supplementary file 1 [file DataSheet_1.docx]

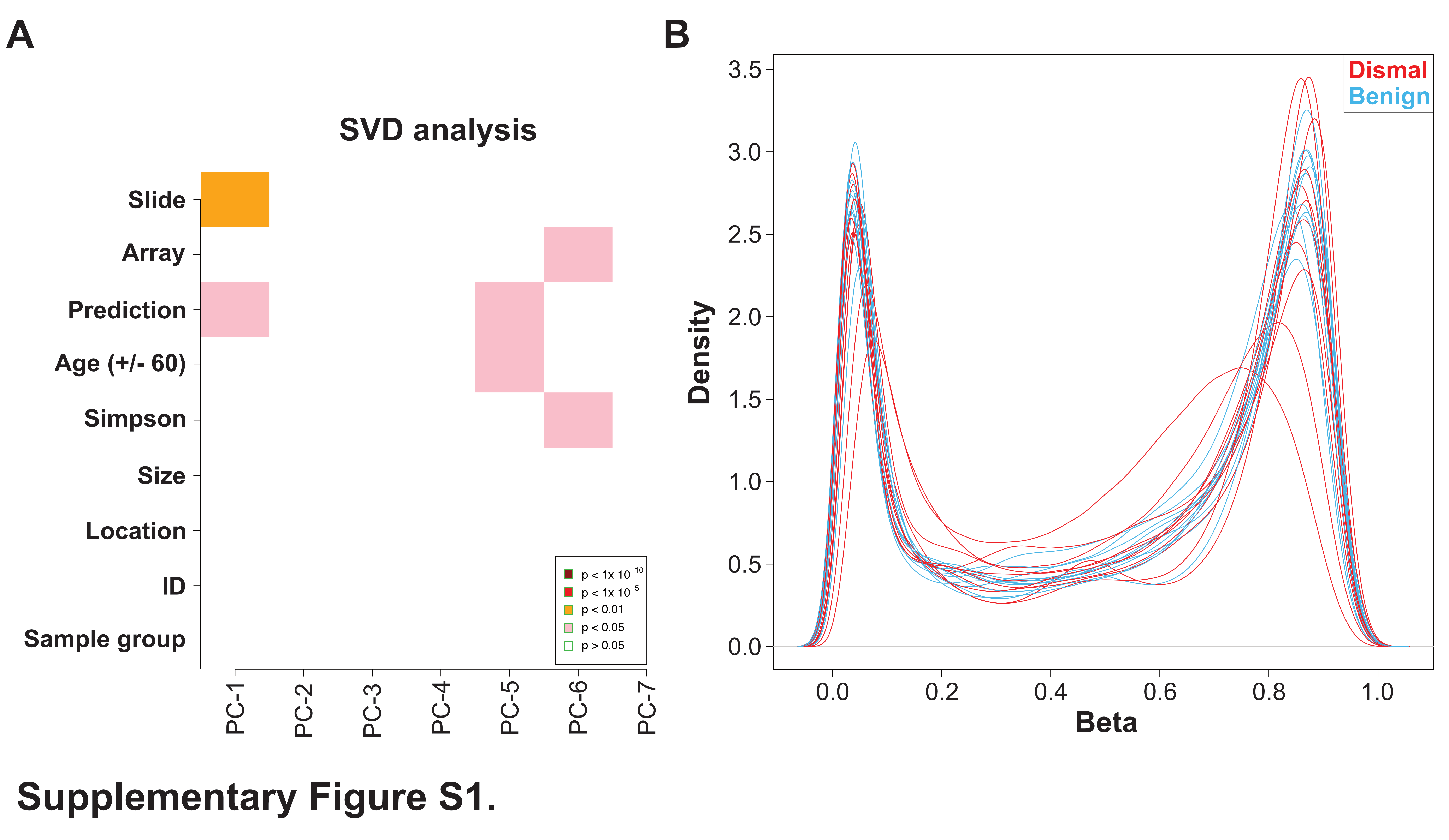


**Supplementary Figure 1.** **(A)** Singular Value Decomposition plot showing the influence of various factors on methylation of the study cohort of 22 meningioma patients. Pink boxes indicate statistical significance (P<0.05). **(B)** Density plot of SWAN normalized beta values of 688,310 probes before batch correction.
